# The international glycan repository GlyTouCan version 3.0

**DOI:** 10.1093/nar/gkaa947

**Published:** 2020-10-30

**Authors:** Akihiro Fujita, Nobuyuki P Aoki, Daisuke Shinmachi, Masaaki Matsubara, Shinichiro Tsuchiya, Masaaki Shiota, Tamiko Ono, Issaku Yamada, Kiyoko F Aoki-Kinoshita

**Affiliations:** Graduate School of Science and Engineering, Soka University, Tokyo 192-8577, Japan; Graduate School of Science and Engineering, Soka University, Tokyo 192-8577, Japan; Graduate School of Science and Engineering, Soka University, Tokyo 192-8577, Japan; The Noguchi Institute, Tokyo 173-0003, Japan; The Noguchi Institute, Tokyo 173-0003, Japan; Graduate School of Science and Engineering, Soka University, Tokyo 192-8577, Japan; Graduate School of Science and Engineering, Soka University, Tokyo 192-8577, Japan; The Noguchi Institute, Tokyo 173-0003, Japan; Graduate School of Science and Engineering, Soka University, Tokyo 192-8577, Japan; Glycan & Life Systems Integration Center (GaLSIC), Faculty of Science and Engineering, Soka University, Tokyo 192-8577, Japan

## Abstract

Glycans serve important roles in signaling events and cell-cell communication, and they are recognized by lectins, viruses and bacteria, playing a variety of roles in many biological processes. However, there was no system to organize the plethora of glycan-related data in the literature. Thus GlyTouCan (https://glytoucan.org) was developed as the international glycan repository, allowing researchers to assign accession numbers to glycans. This also aided in the integration of glycan data across various databases. GlyTouCan assigns accession numbers to glycans which are defined as sets of monosaccharides, which may or may not be characterized with linkage information. GlyTouCan was developed to be able to recognize any level of ambiguity in glycans and uniquely assign accession numbers to each of them, regardless of the input text format. In this manuscript, we describe the latest update to GlyTouCan in version 3.0, its usage, and plans for future development.

## INTRODUCTION

Glycans are carbohydrate sugar chains that can be attached to proteins and lipids on the cell surface. They serve in signaling events and cell–cell communication, and they are recognized by lectins, viruses and bacteria, serving a variety of roles in many biological processes (1). While genes and proteins can be sequenced and computed directly as linear strings, glycans are branched molecules and thus cannot be represented and compared easily. Thus, although many glycan databases have been developed over the years, each used their own independent text representation to store glycan data, making it difficult to exchange and link information with one another (2). This situation prompted the development of a glycan repository, whereby a single accession number could be assigned to glycans published in the literature. Thus GlyTouCan (https://glytoucan.org) was initially released in 2016 as the first and only glycan repository (3).

**Figure 1.**
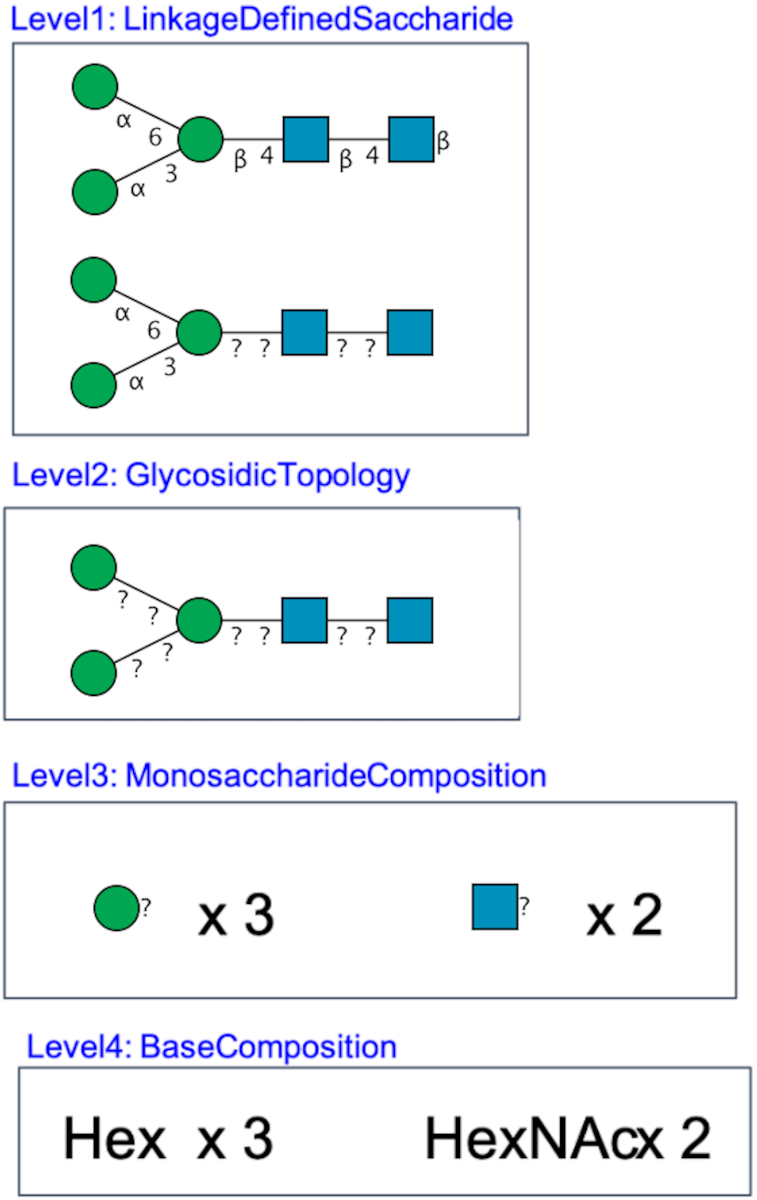
Examples of glycan that can be registered in GlyTouCan. Different levels of ambiguity can be defined.

GlyTouCan assigns accession numbers to glycans which are defined as sets of monosaccharides, which may or may not be characterized with linkage information. For example, Figure 1 is an illustration of various levels of ambiguity when representing glycans, which can range from base compositions to glycans with all linkages fully identified. Because of the current technology for characterizing glycans, publications often report glycans at the base composition or monosaccharide composition level, whereas older publications may have more linkage information defined. Thus, GlyTouCan was developed to be able to recognize any of these levels of glycans and uniquely assign accession numbers to each of them, regardless of the input text format. In this manuscript, we describe the latest updates to GlyTouCan in version 3.0, its usage, and plans for future development.

## MATERIALS AND METHODS

The data in GlyTouCan is based on Semantic Web technologies (4), where all user data and glycan data are stored in a Resource Description Framework (RDF) triplestore. The user interface is developed mainly using Web components, which display data based on queries on the triplestore.

The biggest change in the update to version 3.0 was the registration flow. In the previous version, only GlycoCT (5) and WURCS (6) were the two formats accepted to be registered and assigned a GlyTouCan ID in version 2.0. Registration and assignment of IDs was performed immediately, once simple checks of the format were made to ensure that chemically impossible glycans were not registered. The advantage of this registration flow was that users could obtain accession numbers immediately. The disadvantages were that validation, text format conversion and other calculations were limited to a one-time execution registration process. If these calculations had any issues and needed to be updated, there was no historical context as to exactly how they were previously implemented or changed, for any glycan registered previously or thereafter. Moreover, although extremely rare, the same glycan could be registered within milliseconds of one another, while the previously described calculations were being executed, resulting in the assignment of multiple IDs for the same glycan.

Due to these reasons, GlyTouCan version 3.0 performed a major update to (i) simplify the registration and search functionality and (ii) improve the validity of the content in GlyTouCan. In April 2019, the GlyCosmos Glycoscience Portal (7) was officially released as a web portal to integrate glycan-related omics data. GlyCosmos contains a section of Repositories and one for Data Resources. GlyTouCan, GlycoPOST and most recently UniCarb-DR (8) have become members under the Repositories section. This allowed us to simplify GlyTouCan and move some of the search functionality to GlyCosmos. Therefore, search by mass and motifs, as well as by species, has been removed from GlyTouCan and is now available under Glycan Search in GlyCosmos.

**Figure 2.**
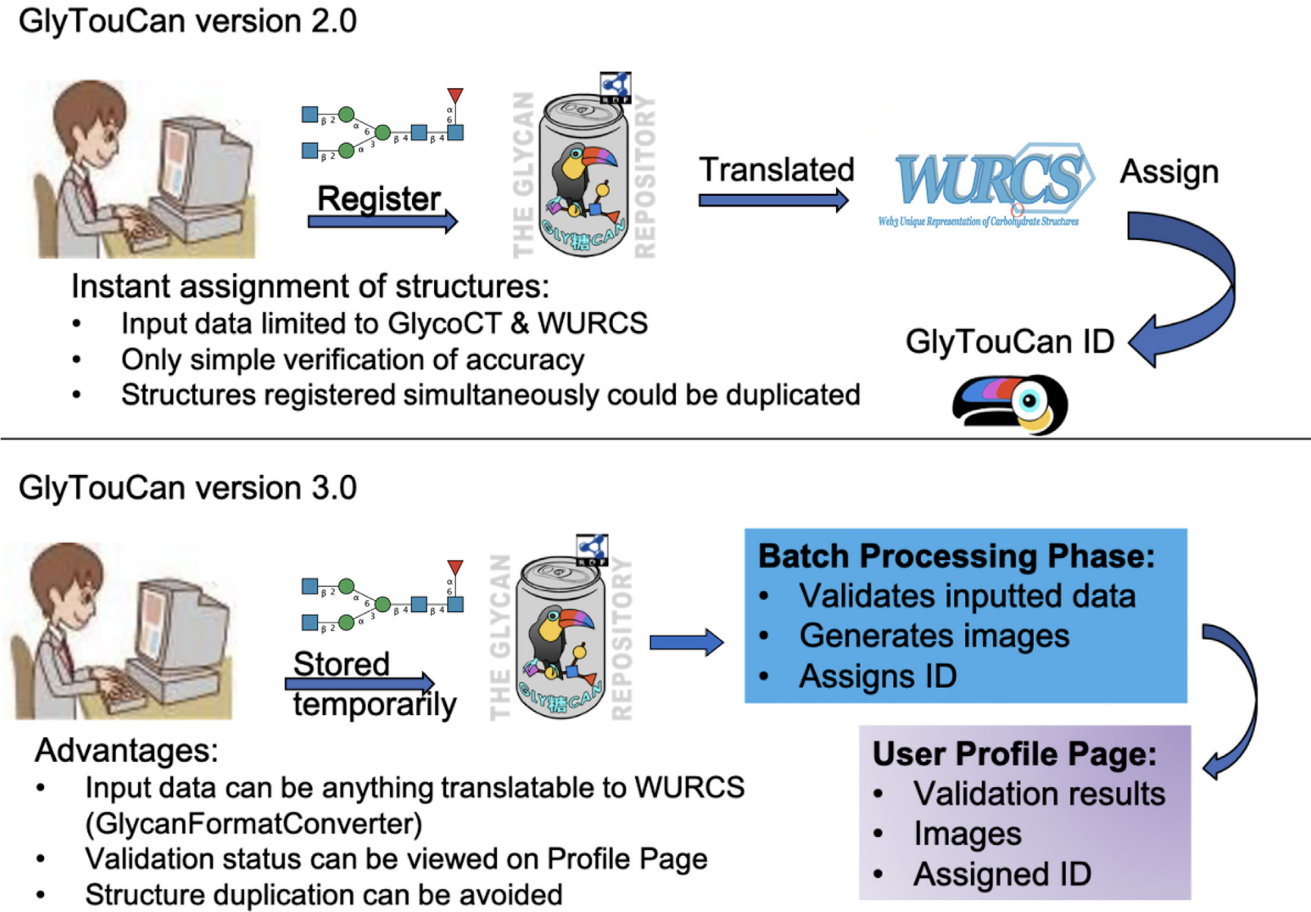
The new registration process in GlyTouCan. The top part represents the former version where registration, translation and assignment of IDs were performed immediately. The bottom represents the new system, where glycans registered are first assigned a hash key and stored in the User's Submissions. A periodic batch process will then validate inputted data, and once validated, generates the glycan image and assigns an accession number. All the inputted data, validation results, generated images and IDs will be listed in the new User's Submissions page.

Figure 2 illustrates the change in the registration process. The top part shows how in the former version, registration, translation and assignment of IDs were performed immediately. The bottom represents the new system, where glycans registered are first assigned a hash key and stored in the User's Submissions page. This submissions page will show the status of the batch processing of the glycans registered. This periodic batch process will validate inputted data, and once validated, generate the glycan image and assign an accession number. All the inputted data, validation results, generated images and IDs will be listed in the new User's Submissions page.

## RESULTS

Here, we illustrate how the new registration process proceeds. Users first login using a Google account. Then the following steps should be taken.

### New registration flow

#### User registration

**Figure 3.**
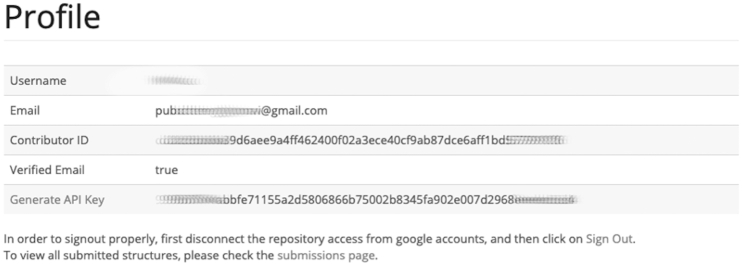
After user registration, the user can check the registration information such as the registered email address, Contributor ID and API Key required for submission of glycans on the user profile page (https://glytoucan.org/Users/profile).

Users must make certain that they have an API key generated in their user profile. This can be generated by clicking the ‘Generate API Key’ link in their user profile. Figure 3 is a screenshot of the user profile page. If no API key is listed, then the ‘Generate API Key’ link should be clicked. The Contributor ID is used for API access to GlyTouCan, so that users can register and search GlyTouCan from a computer program.

#### Glycan data registration

In version 3.0, all data registered in GlyTouCan are initially assigned a hash key on the server, which stores the data, user information and date/time of the registration. This hash key is listed under ‘Submission Ref’ on the user's Data Submissions page.

### Data submissions page

#### User data

**Figure 4.**
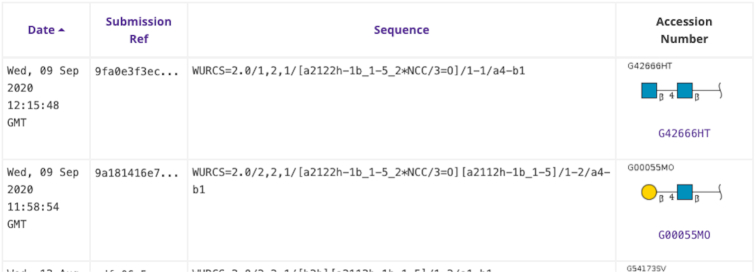
Examples of user-registered glycans and registered information on the Data Submission page (https://glytoucan.org/Users/structure). Initially, the data is sorted based on the date and time of submission (left-most column). Submission Ref is the hash key generated upon registration. Sequence is the user-submitted glycan data. If the batch processing confirms that the data is valid, the accession number and glycan image is displayed in the right-most column. Note that if the glycan was already registered, then the accession number already assigned is listed.

After registering glycan data via the Graphic Tool or by any text format supported by GlyTouCan, users can confirm their submission information on their User's Submissions page (Figure 4). GlycoCT and WURCS are currently the two formats that can be validated, but other formats can be registered and given a reference number; these will be processed once validation tools are available.

### API access

GlyTouCan offers an application programming interface for users to register glycan sequences and to make structured queries to the database. The documentation for the API services is available at https://api.glytoucan.org. Basic authentication over HTTPS is used to transfer user authentication information. Details are available from the online documentation at http://code.glytoucan.org/system/basic/.

### GlyTouCan partner program

The GlyTouCan Partner program is a set of specialized functionality for organizations managing their own glycan information. It primarily allows for the automation of registering glycans, as well as linking back to the partner member's website. The main goal of this program is to expand the Linked Data network (9,10) within the glycobiology community. That is, for those database providers who wish to have a link in GlyTouCan entry pages appear to jump to their own entry pages, manually adding such links will delay the process, especially as more database partners increase. This becomes a full-time process if updates and changes to URLs, etc. arise in different databases. Therefore, this program gives control to the database provider. GlyTouCan provides a special application programming interface to database providers once they register. Then, the database provider can use this interface to make additions, modifications, and deletions of links to particular glycan entries in GlyTouCan.

**Figure 5.**
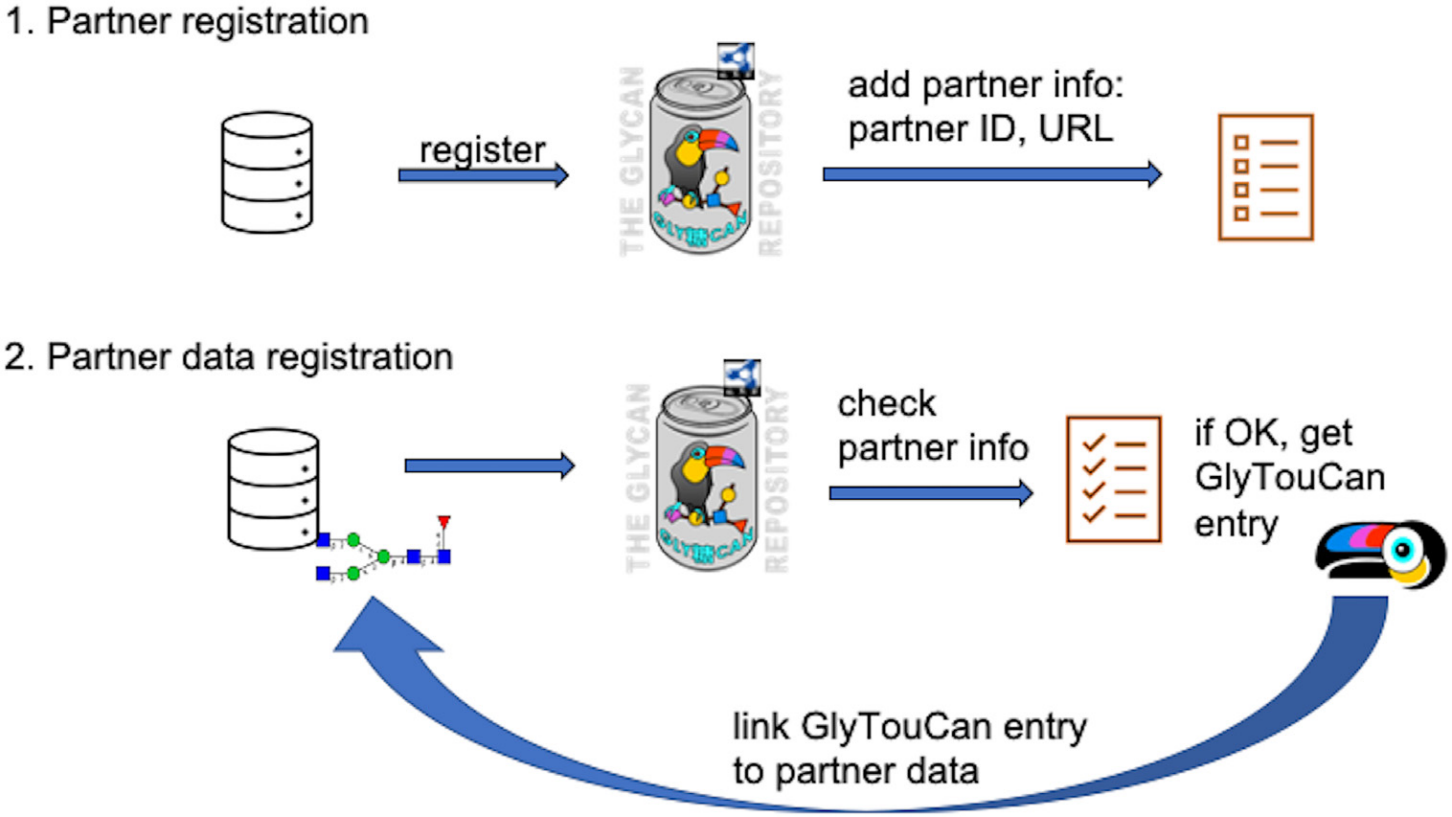
Illustration of the GlyTouCan Partner Program. First, database providers who have glycan data can register as a partner by providing their contributor ID and base URL. Once registered, database provider developers can register their glycans corresponding to GlyTouCan IDs, which will then be checked and, once validated, will generate links in GlyTouCan entry pages back to the partner's corresponding entry page.

Specifically, once the Partner Registration process is complete, GlyTouCan detects whether the user is a partner member. When registering glycans, the partner's glycan id can be submitted along with the glycan data. This will be used with the URL template provided by the partner during the Partner Registration process. The newly registered glycan will then have an entry linking back based on the URL template and ID. By allowing more database providers to easily add links to their database entries from GlyTouCan, users can expect to find more detailed information about their glycans of interest. Figure 5 illustrates the GlyTouCan Partner Program and this process of registering partner IDs with GlyTouCan.

## DISCUSSION

The advantage of this new system is that users are now not limited to submitting glycans in GlycoCT and WURCS formats. Due to the development of GlycanFormatConverter (11), any supported and convertible format can now be registered. Currently, IUPAC condensed and KCF are supported, and more formats are planned in the future. The batch processor will translate the given format into the system's WURCS format in order to assign an accession number.

There were remnants of the former system that required a tedious process of validating the already registered glycans in GlyTouCan. This resulted in the archival of duplicated glycans. A list of the archived entries and the corresponding active entries has been documented. Moreover, GlyCosmos now contains a list of the validated glycans in GlyTouCan, under the GlyCosmos Glycans data resource. Note that since GlyCosmos updates are released every four months, this list is also a reflection of the validated glycans at the time of the latest release. All the archived and active data are available from the GlyCosmos Downloads page.

Most importantly, although there is now an increased waiting time for assignment of accession numbers from the user's perspective, from a system's perspective, the new GlyTouCan system is more stable and able to keep logs of the registration process in more detail. Moreover, the new user's Submissions page makes it easier for users to keep track of the data they have registered. Especially considering the speed at which the glycoinformatics field changes, this new update will enable GlyTouCan to keep up with the latest developments.

## DATA AVAILABILITY

GlyTouCan is available at https://glytoucan.org, and the GlyTouCan triplestore is accessible from the following URL: https://ts.glytoucan.org/sparql. The GlyTouCan API is available at https://api.glytoucan.org/swagger-ui.html. All the archived and active data are available from the GlyCosmos Downloads page at https://glycosmos.org/homes/download.
